# Some Observations on Bone Grafts

**Published:** 1918-08

**Authors:** 


### SOME OBSERVATIONS ON BONE GRAFTS

BY MM. DUFOURMENTEL AND FRISON

We have had occasion to perform bone grafting in seven cases. Four are reported in full in the article; of the other three two are too recent to enable us to say what the result will be; apparently they are going on satisfactorily. The third was a failure, but the conditions under which it was undertaken were so special that the contrary would have been a miracle; it was a question of restoring the entire mandibular arch in a cutaneous sac which itself was reconstituted by grafting.

The large osteo-periostetic graft was partially eliminated by minute fragments through a small fistulous orifice. The remainder was progressively resorbed. No trace of it remains and the periosteum, if there is any, has certainly produced no new element. Having reported the four cases the authors continue: In these four cases we have obtained two perfect results. When we say perfect we mean

(1) That the most critical examination may be made without there resulting the least doubt as to the absolute consolidation.

(2) That mastication is performed normally.

(3) That there does not exist any apparent deformity other than insignificant cicatrices, and in particular the dental articulation is normal.

We must emphasize the importance of intermaxillary immobilization before the graft is put in to ensure this result.

The third case gave us the same satisfaction from the point of view of consolidation, but was marred by an error in occlusion, owing to the fact that the jaws were not fixed to one another, as well as to the fact that the patient's character made him unsuitable for this treatment.

The fourth case is almost completely healed, but functional recuperation has not yet been obtained; we believe it soon will be. It is interesting because the loss of tissue occurred in the region of the angle and ascending ramus where reconstitution by grafting is much more difficult.

If we judge the method by our own cases and by comparison with cartilaginous grafts which have only given us a cosmetic improvement we must conclude that it is excellent. It is simple and has no great inconveniences. Local anesthesia is always sufficient and has the advantage of allowing the fixing of the immobilizing appliance before the graft is put in. The removal of a portion of the tibia is also easier than the removal of a portion of the jaw. The osseous breach is filled up in about a fortnight and no traces remain.

Apparently union has been slow; at the end of two months it did not appear to have begun; at the end of four months it was complete.

We consider that the essential point to decide is: What is the exact role of the graft? Does it form bone? Is it only a feature of union? Is it simply a matrix in the center of a bridge of bone which the fragments themselves will build? Is it, an excitant, or a decoy to the fragments to do their work? Is it, as Sicard and Dambrin believe, the food of the new bone to which it will furnish the chemical elements by its own disorganization?

In spite of our success, we are unable to say; we have indeed seen spontaneous union in some very disturbing cases; some patients who should have been operated on by grafting and have refused have united contrary to all expectation, and after an excessively long time. Skeptical as to the result in these cases, and waiting longer and longer before submitting the patients to grafting we have noticed other delayed and unexpected consolidations.

Herpin has shown us radiographs which clearly indicate in one case which ended in consolidation that the new-formed bridge of bone was developed *by the side of a graft.*

Radiographs do not reveal the formation of a callus independently of the graft, but an incorporation of the former in the latter. We therefore are impressed by the results of grafting in cases which seem to rebel against union, but it is our intention only to perform the operation after prolonged waiting.—*La Restauration Maxillo—Faciale,* Dec., 1917.—*Dental Record.*

